# Reporting guidelines for clinical trial reports for interventions involving artificial intelligence: the CONSORT-AI Extension

**DOI:** 10.1136/bmj.m3164

**Published:** 2020-09-09

**Authors:** Xiaoxuan Liu, Samantha Cruz Rivera, David Moher, Melanie J Calvert, Alastair K Denniston, SPIRIT-AI and CONSORT-AI Working Group, Hutan Ashrafian, Andrew L Beam, An-Wen Chan, Gary S Collins, Ara Darzi, Jonathan J Deeks, M Khair ElZarrad, Cyrus Espinoza, Andre Esteva, Livia Faes, Lavinia Ferrante di Ruffano, John Fletcher, Robert Golub, Hugh Harvey, Charlotte Haug, Christopher Holmes, Adrian Jonas, Pearse A Keane, Christopher J Kelly, Aaron Y Lee, Cecilia S Lee, Elaine Manna, James Matcham, Melissa McCradden, Joao Monteiro, Cynthia Mulrow, Luke Oakden-Rayner, Dina Paltoo, Maria Beatrice Panico, Gary Price, Samuel Rowley, Richard Savage, Rupa Sarkar, Sebastian J Vollmer, Christopher Yau

**Affiliations:** 1 Academic Unit of Ophthalmology, Institute of Inflammation and Ageing, College of Medical and Dental Sciences, University of Birmingham, UK; 2 Department of Ophthalmology, University Hospitals Birmingham NHS Foundation Trust, Birmingham, UK; 3 Moorfields Eye Hospital NHS Foundation Trust, London, UK; 4 Health Data Research UK, London, UK; 5 Birmingham Health Partners Centre for Regulatory Science and Innovation, University of Birmingham, Birmingham, UK; 6 Centre for Patient Reported Outcome Research, Institute of Applied Health Research, University of Birmingham, Birmingham, UK; 7 Centre for Journalology, Clinical Epidemiology Program, Ottawa Hospital Research Institute, Ottawa, Canada; 8 School of Epidemiology and Public Health, University of Ottawa, Ottawa, Canada; 9 National Institute of Health Research Surgical Reconstruction and Microbiology Centre, University of Birmingham and University Hospitals Birmingham NHS Foundation Trust, Birmingham, UK; 10 National Institute of Health Research Birmingham Biomedical Research Centre, University of Birmingham and University Hospitals Birmingham NHS Foundation Trust, Birmingham, UK; 11 National Institute of Health Research Applied Research Collaborative West Midlands, Birmingham, UK; 12 National Institute of Health Research Biomedical Research Centre for Ophthalmology, Moorfields Hospital London NHS Foundation Trust and University College London, Institute of Ophthalmology, London, UK

## Abstract

The CONSORT 2010 (Consolidated Standards of Reporting Trials) statement provides minimum guidelines for reporting randomised trials. Its widespread use has been instrumental in ensuring transparency when evaluating new interventions. More recently, there has been a growing recognition that interventions involving artificial intelligence (AI) need to undergo rigorous, prospective evaluation to demonstrate impact on health outcomes.

The CONSORT-AI extension is a new reporting guideline for clinical trials evaluating interventions with an AI component. It was developed in parallel with its companion statement for clinical trial protocols: SPIRIT-AI. Both guidelines were developed through a staged consensus process, involving a literature review and expert consultation to generate 29 candidate items, which were assessed by an international multi-stakeholder group in a two-stage Delphi survey (103 stakeholders), agreed on in a two-day consensus meeting (31 stakeholders) and refined through a checklist pilot (34 participants).

The CONSORT-AI extension includes 14 new items, which were considered sufficiently important for AI interventions, that they should be routinely reported in addition to the core CONSORT 2010 items. CONSORT-AI recommends that investigators provide clear descriptions of the AI intervention, including instructions and skills required for use, the setting in which the AI intervention is integrated, the handling of inputs and outputs of the AI intervention, the human-AI interaction and providing analysis of error cases.

CONSORT-AI will help promote transparency and completeness in reporting clinical trials for AI interventions. It will assist editors and peer-reviewers, as well as the general readership, to understand, interpret and critically appraise the quality of clinical trial design and risk of bias in the reported outcomes.

## Introduction

Randomised controlled trials (RCTs) are considered the gold-standard experimental design to provide evidence of the safety and efficacy of an intervention.^1^ ^2^ Trial results, if adequately reported, have the potential to inform regulatory decisions, clinical guidelines and health policy. It is therefore crucial that RCTs are reported with transparency and completeness, so that readers can critically appraise the trial methods and findings and assess for the presence of bias in the results.^3^ ^4^ ^5^

The CONSORT (Consolidated Standards of Reporting Trials) statement provides evidence-based recommendations to improve the completeness of reporting of RCTs. The statement was first introduced in 1996 and has since been widely endorsed by medical journals internationally.^5^ Over the last two decades, it has undergone two updates and has demonstrated a significant positive impact on the quality of RCT reports.^6^ ^7^ The most recent CONSORT 2010 statement provides a 25 item checklist of the minimum reporting content applicable to all RCTs, but recognises that certain interventions may require extension or elaboration of these items. Several such extensions exist.^8^ ^9^ ^10^ ^11^ ^12^ ^13^

Artificial intelligence (AI) is an area of enormous interest with strong drivers to accelerate new interventions through to publication, implementation and market.^14^ While AI systems have been researched for some time, recent advances in deep learning and neural networks have gained significant interest for their potential in health applications. Examples of such applications are wide-ranging and include AI systems for screening and triage,^15^ ^16^ diagnosis,^17^ ^18^ ^19^ ^20^prognostication,^21^ ^22^ decision-support^23^ and treatment recommendation.^24^ However, in most recent cases, published evidence consists of *in silico*, early-phase validation. It has been recognised that most recent AI studies are inadequately reported and existing reporting guidelines do not fully cover potential sources of bias specific to AI systems.^25^ The welcome emergence of randomised controlled trials (RCTs) seeking to evaluate newer interventions based on, or including, an AI component (hereafter “AI interventions”)^23^ ^26^ ^27^ ^28^ ^29^ ^30^ ^31^ has similarly been met with concerns about the design and reporting.^25^ ^32^ ^33^ ^34^ This has highlighted the need to provide reporting guidance that is “fit-for-purpose” in this domain.

CONSORT-AI (as part of the SPIRIT-AI & CONSORT-AI initiative) is an international initiative supported by CONSORT and the EQUATOR Network to evaluate the existing CONSORT 2010 statement and extend or elaborate this guidance where necessary, to support reporting of clinical trials for AI-interventions.^35^ ^36^ It is complementary to the SPIRIT (Standard Protocol Items: Recommendations for Interventional Trials)-AI statement, which aims to promote high quality protocol reporting for AI trials. This article describes the methods used to identify and evaluate candidate items and gain consensus. In addition, it also provides the CONSORT-AI checklist, which includes the new extension items and their accompanying explanations.

## Methods

The SPIRIT-AI and CONSORT-AI extensions were simultaneously developed for clinical trial protocols and trial reports. An announcement for the SPIRIT-AI and CONSORT-AI initiative was published in October 2019,^35^ and the two guidelines were registered as reporting guidelines under development on the EQUATOR library of reporting guidelines in May 2019. Both guidelines were developed in accordance with the EQUATOR Network’s methodological framework.^37^ The SPIRIT-AI and CONSORT-AI steering group, consisting of 15 international experts, was formed to oversee the conduct and methodology of the study. Definitions of key terms are contained in the glossary box 1.

Box 1 **Glossary**

*Artificial intelligence (AI)*—The science of developing computer systems which can perform tasks normally requiring human intelligence.
*AI intervention*—A health intervention which relies on an artificial intelligence/machine learning component to serve its purpose.
*CONSORT*—Consolidated Standards of Reporting Trials.
*CONSORT-AI extension item*—An additional checklist item to address AI-specific content that is not adequately covered by CONSORT 2010.
*Class activation map*—Class activation maps are particularly relevant to image classification AI interventions. Class activation maps are visualizations of the pixels that had the greatest influence on predicted class, by displaying the gradient of the predicted outcome from the model with respect to the input. They are also referred to as saliency maps or heatmaps.
*Health outcome*—Measured variables in the trial which are used to assess the effects of an intervention.
*Human-AI interaction*—The process of how users/humans interact with the AI intervention, for the AI intervention to function as intended.
*Clinical outcome*—Measured variables in the trial which are used to assess the effects of an intervention.
*Delphi study*—A research method which derives the collective opinions of a group through a staged consultation of surveys, questionnaires, or interviews, with an aim to reach consensus at the end.
*Development environment*—The clinical and operational settings from which the data used for training the model is generated. This includes all aspects of the physical setting (such as geographical location, physical environment), operational setting (such as integration with an electronic record system, installation on a physical device) and clinical setting (such as primary/secondary/tertiary care, patient disease spectrum).
*Fine-tuning*—Modifications or additional training performed on the AI intervention model, done with the intention of improving its performance.
*Input data*—The data that need to be presented to the AI intervention to allow it to serve its purpose.
*Machine learning (ML)*—A field of computer science concerned with the development of models/algorithms which can solve specific tasks by learning patterns from data, rather than by following explicit rules. It is seen as an approach within the field of artificial intelligence.
*Operational environment*—The environment in which the AI intervention will be deployed, including the infrastructure required to enable the AI intervention to function.
*Output data*—The predicted outcome given by the AI intervention based on modelling of the input data. The output data can be presented in different forms, including a classification (including diagnosis, disease severity or stage, or recommendation such as referability), a probability, a class activation map, etc. The output data typically provides additional clinical information and/or triggers a clinical decision.
*Performance error*—Instances where the AI intervention fails to perform as expected. This term can describe different types of failures and it is up to the investigator to specify what should be considered a performance error, preferably based on prior evidence. This can range from small decreases in accuracy (compared to expected accuracy), to erroneous predictions, or the inability to produce an output in certain cases.
*SPIRIT*—Standard Protocol Items: Recommendations for Interventional Trials.
*SPIRIT-AI*—An additional checklist item to address AI-specific content that is not adequately covered by SPIRIT 2013.
*SPIRIT-AI elaboration item*—Additional considerations to an existing SPIRIT 2013 item when applied to AI interventions.

### Ethical approval

This study was approved by the ethical review committee at the University of Birmingham, UK (ERN_19-1100). Participant information was provided to Delphi participants electronically prior to survey completion and prior to the consensus meeting. Delphi participants provided electronic informed consent, and written consent was obtained from consensus meeting participants.

### Literature review and candidate item generation

An initial list of candidate items for the SPIRIT-AI and CONSORT-AI checklists was generated through review of the published literature and consultation with the steering group and known international experts. A search was performed on 13th May 2019 using the terms “artificial intelligence,” “machine learning” and “deep learning” to identify existing clinical trials for AI interventions listed within the US National Library of Medicine’s clinical trial registry, ClinicalTrials.gov. There were 316 registered trials on ClinicalTrials.gov, of which 62 were completed and seven had published results.^30^ ^38^ ^39^ ^40^ ^41^ ^42^ ^43^ Two studies were reported with reference to the CONSORT statement^30^ ^42^ and one study provided an unpublished trial protocol.^42^ The Operations Team (XL, SCR, MJC and AKD) identified AI-specific considerations from these studies and reframed them as candidate reporting items. The candidate items were also informed by findings from a previous systematic review which evaluated the diagnostic accuracy of deep learning systems for medical imaging.^25^ After consultation with the steering group and additional international experts (n=19), 29 candidate items were generated: 26 of which were relevant for both SPIRIT-AI and CONSORT-AI and three of which were relevant only for CONSORT-AI. The Operations Team mapped these items to the corresponding SPIRIT and CONSORT items, revising the wording and providing explanatory text as required to contextualise the items. These items were included in subsequent Delphi surveys.

### Delphi consensus process

In September 2019, 169 key international experts were invited to participate in the online Delphi survey to vote on the candidate items and suggest additional items. Experts were identified and contacted via the steering group and were allowed one round of snowball recruitment, where contacted experts could suggest additional experts. In addition, individuals who made contact following publication of the announcement were included.^35^ The steering group agreed that individuals with expertise in clinical trials and AI/ML, as well as key users of the technology should be well represented in the consultation. Stakeholders included healthcare professionals, methodologists, statisticians, computer scientists, industry representatives, journal editors, policy makers, health informaticists, law and ethicists, regulators, patients and funders. Participant characteristics are described in the appendix (page 1: supplementary table 1). Two online Delphi surveys were conducted. DelphiManager software (version 4.0), developed and maintained by the COMET (Core Outcome Measures in Effectiveness Trials) initiative, was used to undertake the e-Delphi survey. Participants were given written information about the study and asked to provide their level of expertise within the fields of (i) AI/ML, and (ii) clinical trials. Each item was presented for consideration (26 for SPIRIT-AI and 29 for CONSORT-AI). Participants were asked to vote on each item using a 9-point scale: (1-3) not important, (4-6) important but not critical, and (7-9) important and critical. Respondents provided separate ratings for SPIRIT-AI and CONSORT-AI. There was an option to opt out of voting for each item, and each item included space for free text comments. At the end of the Delphi survey, participants had the opportunity to suggest new items. One hundred and three responses were received for the first Delphi round, and 91 (88% of participants from round one) responses received for the second round. The results of the Delphi survey informed the subsequent international consensus meeting. Twelve new items were proposed by the Delphi study participants and were added for discussion at the consensus meeting. Data collected during the Delphi survey were anonymised and item-level results were presented at the consensus meeting for discussion and voting.

The two-day consensus meeting took place in January 2020 and was hosted by the University of Birmingham, UK, to seek consensus on the content of SPIRIT-AI and CONSORT-AI. Thirty one international stakeholders were invited from the Delphi survey participants to discuss the items and vote for their inclusion. Participants were selected to achieve adequate representation from all the stakeholder groups. Forty one items were discussed in turn, comprising the 29 items generated in the initial literature review and item generation phase (26 items relevant to both SPIRIT-AI and CONSORT-AI; three items relevant to CONSORT-AI only) and the 12 new items proposed by participants during the Delphi surveys. Each item was presented to the consensus group, alongside its score from the Delphi exercise (median and interquartile ranges) and any comments made by Delphi participants related to that item. Consensus meeting participants were invited to comment on the importance of each item and whether the item should be included in the AI extension. In addition, participants were invited to comment on the wording of the explanatory text accompanying each item and the position of each item relative to the SPIRIT 2013 and CONSORT 2010 checklists. After open discussion of each item and the option to adjust wording, an electronic vote took place with the option to include or exclude the item. An 80% threshold for inclusion was pre-specified and deemed reasonable by the steering group to demonstrate majority consensus. Each stakeholder voted anonymously using Turning Point voting pads (Turning Technologies LLC, Ohio, USA; version 8.7.2.14).

### Checklist pilot

Following the consensus meeting, attendees were given the opportunity to make final comments on the wording and agree that the updated SPIRIT-AI and CONSORT-AI items reflected discussions from the meeting. The Operations Team assigned each item as extension or elaboration based on a decision tree and produced a penultimate draft of the SPIRIT-AI and CONSORT-AI checklist (supplementary fig 1 on bmj.com). A pilot of the penultimate checklist was conducted with 34 participants to ensure clarity of wording. Experts participating in the pilot included: a) Delphi participants who did not attend the consensus meeting and b) external experts, who had not taken part in the development process, but who had reached out to the steering committee after the Delphi study commenced. Final changes were made on wording only to improve clarity for readers, by the Operations Team (supplementary fig 2).

## Results

### CONSORT-AI checklist items and explanations

The CONSORT-AI Extension recommends that 14 new checklists items are added to the existing CONSORT 2010 statement (11 extensions and three elaborations). These items were considered sufficiently important for clinical trial reports for AI interventions that they should be routinely reported in addition to the core CONSORT 2010 checklist items. Table 1 lists the CONSORT-AI items.

**Table 1:** CONSORT-AI checklist

Section	Item	CONSORT 2010 item*	CONSORT-AI item		Addressed on page No†
**Title and abstract**					
Title and abstract	1a	Identification as a randomised trial in the title	CONSORT-AI 1a,b Elaboration	(i) Indicate that the intervention involves artificial intelligence/machine learning in the title and/or abstract and specify the type of model.	
	1b	Structured summary of trial design, methods, results, and conclusions (for specific guidance see CONSORT for abstracts)		(ii) State the intended use of the AI intervention within the trial in the title and/or abstract.	
**Introduction**					
Background and objectives	2a	Scientific background and explanation of rationale	CONSORT-AI 2a (i) Extension	Explain the intended use of the AI intervention in the context of the clinical pathway, including its purpose and its intended users (e.g. healthcare professionals, patients, public).	
	2b	Specific objectives or hypotheses			
**Methods**					
Trial design	3a	Description of trial design (such as parallel, factorial) including allocation ratio			
	3b	Important changes to methods after trial commencement (such as eligibility criteria), with reasons			
Participants	4a	Eligibility criteria for participants	CONSORT-AI 4a (i) Elaboration	State the inclusion and exclusion criteria at the level of participants.	
			CONSORT-AI 4a (ii) Extension	State the inclusion and exclusion criteria at the level of the input data.	
	4b	Settings and locations where the data were collected	CONSORT-AI 4b Extension	Describe how the AI intervention was integrated into the trial setting, including any onsite or offsite requirements.	
Interventions	5	The interventions for each group with sufficient details to allow replication, including how and when they were actually administered	CONSORT-AI 5 (i) Extension	State which version of the AI algorithm was used.	
			CONSORT-AI 5 (ii) Extension	Describe how the input data were acquired and selected for the AI intervention.	
			CONSORT-AI 5 (iii) Extension	Describe how poor quality or unavailable input data were assessed and handled.	
			CONSORT-AI 5 (iv) Extension.	Specify whether there was human-AI interaction in the handling of the input data, and what level of expertise was required of users.	
			CONSORT-AI 5 (v) Extension	Specify the output of the AI intervention	
			CONSORT-AI 5 (vi) Extension	Explain how the AI intervention’s outputs contributed to decision-making or other elements of clinical practice.	
Outcomes	6a	Completely defined pre-specified primary and secondary outcome measures, including how and when they were assessed			
	6b	Any changes to trial outcomes after the trial commenced, with reasons			
Sample size	7a	How sample size was determined			
	7b	When applicable, explanation of any interim analyses and stopping guidelines			
**Randomisation**					
Sequence generation	8a	Method used to generate the random allocation sequence			
	8b	Type of randomisation; details of any restriction (such as blocking and block size)			
Allocation concealment mechanism	9	Mechanism used to implement the random allocation sequence (such as sequentially numbered containers), describing any steps taken to conceal the sequence until interventions were assigned			
Implementation	10	Who generated the random allocation sequence, who enrolled participants, and who assigned participants to interventions			
Blinding	11a	If done, who was blinded after assignment to interventions (for example, participants, care providers, those assessing outcomes) and how			
	11b	If relevant, description of the similarity of interventions			
Statistical methods	12a	Statistical methods used to compare groups for primary and secondary outcomes			
	12b	Methods for additional analyses, such as subgroup analyses and adjusted analyses			
**Results**					
Participant flow (a diagram is strongly recommended)	13a	For each group, the numbers of participants who were randomly assigned, received intended treatment, and were analysed for the primary outcome			
	13b	For each group, losses and exclusions after randomisation, together with reasons			
Recruitment	14a	Dates defining the periods of recruitment and follow-up			
	14b	Why the trial ended or was stopped			
Baseline data	15	A table showing baseline demographic and clinical characteristics for each group			
Numbers analysed	16	For each group, number of participants (denominator) included in each analysis and whether the analysis was by original assigned groups			
Outcomes and estimation	17a	For each primary and secondary outcome, results for each group, and the estimated effect size and its precision (such as 95% confidence interval)			
	17b	For binary outcomes, presentation of both absolute and relative effect sizes is recommended			
Ancillary analyses	18	Results of any other analyses performed, including subgroup analyses and adjusted analyses, distinguishing pre-specified from exploratory			
Harms	19	All important harms or unintended effects in each group (for specific guidance see CONSORT for harms)	CONSORT-AI 19 Extension	Describe results of any analysis of performance errors and how errors were identified, where applicable. If no such analysis was planned or done, justify why not.	
**Discussion**					
Limitations	20	Trial limitations, addressing sources of potential bias, imprecision, and, if relevant, multiplicity of analyses			
Generalisability	21	Generalisability (external validity, applicability) of the trial findings			
Interpretation	22	Interpretation consistent with results, balancing benefits and harms, and considering other relevant evidence			
**Other information**					
Registration	23	Registration number and name of trial registry			
Protocol	24	Where the full trial protocol can be accessed, if available			
Funding	25	Sources of funding and other support (such as supply of drugs), role of funders	CONSORT-AI 25 Extension.	State whether and how the AI intervention and/or its code can be accessed, including any restrictions to access or re-use.	

* We strongly recommend reading this statement in conjunction with the CONSORT 2010 Explanation and Elaboration for important clarifications on all the items.

† Indicates page numbers to be completed by authors during protocol development.

The 14 items below passed the threshold of 80% for inclusion at the consensus meeting. CONSORT-AI 2a, CONSORT-AI 5 (ii), and CONSORT-AI 19 each resulted from the merging of two items after discussion with the consensus group. CONSORT-AI 4a (i) and (ii) was split into two items for clarity and voted on separately. CONSORT-AI 5(iii) did not fulfill the criteria for inclusion based on its initial wording (77% vote to include); however, after extensive discussion and rewording, the consensus group unanimously supported a re-vote at which point it passed the inclusion threshold (97% to include). The Delphi and voting results for each included and excluded item are described in the appendix (page 2: supplementary table 2).

### Title and abstract

#### CONSORT-AI 1a,b (i) Elaboration: Indicate that the intervention involves artificial intelligence/machine learning in the title and/or abstract and specify the type of model.

*Explanation:* Indicating in the title and/or abstract of the trial report that the intervention involves a form of AI is encouraged, as it immediately identifies the intervention as an artificial intelligence/machine learning intervention and also serves to facilitate indexing and searching of the trial report. The title should be understandable by a wide audience, therefore a broader umbrella term such as artificial intelligence or machine learning is encouraged. More precise terms should be used in the abstract, rather than the title, unless broadly recognised as being a form of artificial intelligence/machine learning. Specific terminology relating to the model type and architecture should be detailed in the abstract.

#### CONSORT-AI 1a,b (ii) Elaboration: State the intended use of the AI intervention within the trial in the title and/or abstract.

*Explanation:* Describe the intended use of the AI intervention in the trial report title and/or abstract. This should describe the purpose of the AI intervention and the disease context.^26^ ^44^ Some AI interventions may have multiple intended uses or the intended use may evolve over time. Therefore, documenting this allows readers to understand the intended use of the algorithm at the time of the trial.

### Introduction

#### CONSORT-AI 2a (i) Extension: Explain the intended use for the AI intervention in the context of the clinical pathway, including its purpose and its intended users (such as healthcare professionals, patients, public).

*Explanation:* In order to understand how the AI intervention is intended to fit into a clinical pathway, a detailed description of its role should be included in the background of the trial report. AI interventions may be designed to interact with different users including healthcare professionals, patients and the public, and its role can be wide-ranging (for example, the same AI intervention could theoretically be replacing, augmenting, or adjudicating components of clinical decision-making). Clarifying the intended use of the AI intervention and its intended user helps readers understand the purpose for which the AI intervention was evaluated in the trial.

### Methods

#### CONSORT-AI 4a (i) Elaboration: State the inclusion and exclusion criteria at the level of participants.

*Explanation:* The inclusion and exclusion criteria should be defined at the participant level as per usual practice in non-AI interventional trial reports. This is distinct from the inclusion and exclusion criteria made at the input data level, which is addressed in item 4a (ii).

#### CONSORT-AI 4a (ii) Extension: State the inclusion and exclusion criteria at the level of the input data.

**Fig 1:**
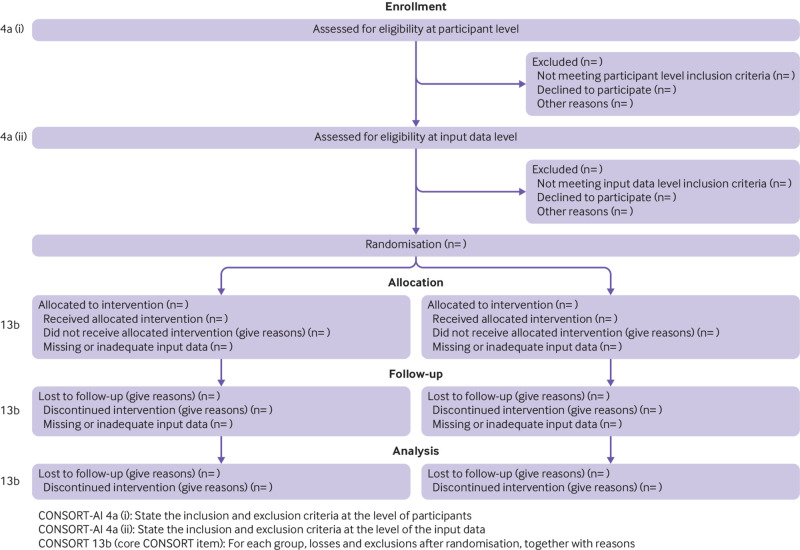
CONSORT 2010 flow diagram—adapted for AI clinical trials

*Explanation:* Input data refer to the data required by the AI intervention to serve its purpose (for example, for a breast cancer diagnostic system, the input data could be the unprocessed or vendor-specific post-processing mammography scan on which a diagnosis is being made; for an early warning system, the input data could be physiological measurements or laboratory results from the electronic health record). The trial report should pre-specify if there were minimum requirements for the input data (such as image resolution, quality metrics or data format) which determined pre-randomisation eligibility. It should specify when, how, and by whom this was assessed. For example, if a participant met the eligibility criteria for lying flat for a CT scan as per item 4a (i), but the scan quality was compromised (for any given reason) to such a level that it was deemed unfit for use by the AI system, this should be reported as an exclusion criterion at the input data level. Note that where input data are acquired after randomisation, any exclusion is considered to be from the analysis, not from enrolment (see CONSORT item 13b and fig 1).

#### CONSORT-AI 4b Extension: Describe how the AI intervention was integrated into the trial setting, including any onsite or offsite requirements.

*Explanation:* There are limitations to the generalisability of AI algorithms, one of which is when they are used outside of their development environment.^45^ ^46^ AI systems are dependent on their operational environment and the report should provide details of the hardware and software requirements to allow technical integration of the AI intervention at each study site. For example, it should be stated if the AI intervention required vendor-specific devices, if there was specialised computing hardware at each site, or if the site had to support cloud integration, particularly if this was vendor-specific. If any changes to the algorithm were required at each study site as part of the implementation procedure (such as fine-tuning the algorithm on local data), then this process should also be clearly described.

#### CONSORT-AI 5 (i) Extension: State which version of the AI algorithm was used.

*Explanation:* Similar to other forms of software as a medical device, AI systems are likely to undergo multiple iterations and updates in their lifespan. It is therefore important to specify which version of the AI system was used in the clinical trial, whether this is the same as the version evaluated in previous studies that have been used to justify the study rationale, and whether the version changed during the conduct of the trial. If applicable, the report should describe what has changed between the relevant versions and the rationales for the changes. Where available, the report should include a regulatory marking reference, such as a unique device identifier (UDI) which requires a new identifier for updated versions of the device.^47^

#### CONSORT-AI 5 (ii) Extension: Describe how the input data were acquired and selected for the AI intervention.

*Explanation:* The measured performance of any AI system may be critically dependent on the nature and quality of the input data.^48^ A description of the input data handling, including acquisition, selection, and pre-processing prior to analysis by the AI system should be provided. Completeness and transparency of this description is integral to the replicability of the intervention beyond the clinical trial in real-world settings. It also helps readers identify whether input data handling procedures were standardised across trial sites.

#### CONSORT-AI 5 (iii) Extension: Describe how poor quality or unavailable input data were assessed and handled.

*Explanation:* As with 4a (ii), input data refer to the data required by the AI intervention to serve its purpose. As discussed in CONSORT-AI 4a (ii), the performance of AI systems may be compromised as a result of poor quality or missing input data^49^ (for example, excessive movement artefact on an electrocardiogram). The trial report should report the amount of missing data, as well as how this was identified and handled. The report should also specify if there was a minimum standard required for the input data, and where this standard was not achieved, how this was handled (including the impact on, or any changes to, the participant care pathway).

Poor quality or unavailable data can also affect non-AI interventions. For example, suboptimal quality of a scan could impact a radiologist’s ability to interpret it and make a diagnosis. It is therefore important that this information is reported equally in the control intervention, where relevant. If this minimum quality standard was different from the inclusion criteria for input data used to assess eligibility pre-randomisation, this should be stated.

#### CONSORT-AI 5 (iv) Extension: Specify whether there was human-AI interaction in the handling of the input data, and what level of expertise was required of users.

*Explanation:* A description of the human-AI interface and the requirements for successful interaction when handling input data should be described. For example, clinician-led selection of regions of interest from a histology slide which is then interpreted by an AI diagnostic system,^50^ or endoscopist selection of a colonoscopy video clips as input data for an algorithm designed to detect polyps.^28^ A description of any user training provided and instructions for how users should handle the input data provides transparency and replicability of trial procedures. Poor clarity on the human-AI interface may lead to lack of a standard approach and carry ethical implications, particularly in the event of harm.^51^ ^52^ For example, it may become unclear whether an error case occurred due to human deviation from the instructed procedure, or if it was an error made by the AI system.

#### CONSORT-AI 5 (v) Extension: Specify the output of the AI intervention

*Explanation:* The output of the AI intervention should be clearly specified in the trial report. For example, an AI system may output a diagnostic classification or probability, a recommended action, an alarm alerting to an event, an instigated action in a closed-loop system (such as titration of drug infusions), or other. The nature of the AI intervention's output has direct implications on its usability and how it may lead to downstream actions and outcomes.

#### CONSORT-AI 5 (vi) Extension: Explain how the AI intervention’s outputs contributed to decision-making or other elements of clinical practice.

*Explanation:* Since health outcomes may also critically depend on how humans interact with the AI intervention, the report should explain how the outputs of the AI system were used to contribute to decision-making or other elements of clinical practice. This should include adequate description of downstream interventions which can impact outcomes. As with CONSORT-AI 5 (iv), any elements of human-AI interaction on the outputs should be described in detail, including the level of expertise required to understand the outputs and any training/instructions provided for this purpose. For example, a skin cancer detection system that produced a percentage likelihood as output should be accompanied by an explanation of how this output was interpreted and acted on by the user, specifying both the intended pathways (such as skin lesion excision if the diagnosis is positive) and the thresholds for entry to these pathways (such as skin excision if the diagnosis is positive and the probability is >80%). The information produced by comparator interventions should be similarly described, alongside an explanation of how such information was used to arrive at clinical decisions on patient management, where relevant. Any discrepancy in how decision-making occurred versus how it was intended to occur (that is, as specified in the trial protocol), should be reported.

### Results

#### CONSORT-AI 19 Extension: Describe results of any analysis of performance errors and how errors were identified, where applicable. If no such analysis was planned or done, explain why not.

*Explanation:* Reporting performance errors and failure case analysis is especially important for AI interventions. AI systems can make errors that may be hard to foresee, but which, if allowed to be deployed at scale, could have catastrophic consequences.^53^ Therefore, reporting cases of error and defining risk mitigation strategies are important for informing when, and for which populations, the intervention can be safely implemented. The results of any performance error analysis should be reported and the implications of the results discussed.

### Other information

#### CONSORT-AI 25 Extension: State whether and how the AI intervention and/or its code can be accessed, including any restrictions to access or re-use.

*Explanation:* The trial report should make it clear whether and how the AI intervention and/or its code can be accessed or re-used. This should include details regarding the license and any restrictions to access.

## Discussion

CONSORT-AI is a new reporting guideline extension developed through international multi-stakeholder consensus. It aims to promote transparent reporting of AI intervention trials and is intended to facilitate critical appraisal and evidence synthesis. The extension items added in CONSORT-AI address a number of issues specific to the implementation and evaluation of AI interventions, which should be considered alongside the core CONSORT 2010 checklist and other CONSORT extensions.^54^ It is important to note that these are minimum requirements and there may be value in including additional items not included in the checklists (see appendix, page 2: supplementary table 2) in the report or in supplementary materials.

In both CONSORT-AI and its companion project SPIRIT-AI, a major emphasis was the addition of several new items relating to the intervention itself and its application in the clinical context. Items 5 (i) to 5 (vi) were added to address AI-specific considerations when describing the intervention. Specific recommendations were made pertinent to AI systems relating to algorithm version, input and output data, integration into trial settings, expertise of the users, and protocol for acting on the AI system’s recommendations. It was agreed that these details are critical for independent evaluation or replication of the trial. Journal editors reported that, despite the importance of these items, they are currently often missing from trial reports at the time of submission for publication, providing further weight to their inclusion as specifically listed extension items.

A recurrent focus of the Delphi comments and consensus group discussion was around safety of AI systems. This was in recognition that AI systems, unlike other health interventions, can unpredictably yield errors which are not easily detectable or explainable by human judgment. For example, changes to medical imaging that are invisible or appear random to the human eye may change the likelihood of the diagnostic output entirely.^55^ ^56^ The concern is, given the theoretical ease at which AI systems could be deployed at scale, any unintended harmful consequences could be catastrophic. CONSORT-AI item 19, which requires specification of any plans to analyse performance errors was added to emphasise the importance of anticipating systematic errors made by the algorithm and their consequences. Beyond this, investigators should also be encouraged to explore differences in performance and error rates across population subgroups. It has been shown that AI systems may be systematically biased towards different outputs, which may lead to different or even unfair treatment on the basis of extant features.^53^ ^57^ ^58^ ^59^

The topic of “continuously evolving” AI systems (also known as “continuously adapting” or “continuously learning”) was discussed at length during the consensus meeting, but was agreed to be excluded from CONSORT-AI. These are AI systems with the ability to continuously train on new data, which may cause changes in performance over time. The group noted that, while of interest, this field is relatively early in its development without tangible examples in healthcare applications, and that it would not be appropriate for it to be included in CONSORT-AI at this stage.^60^ This topic will be monitored and revisited in future iterations of CONSORT-AI. It is worth noting that incremental software changes, whether continuous or iterative, intentional or unintentional, could have serious consequences on safety performance after deployment. It is therefore of vital importance that such changes are documented and identified by software version and a robust post-deployment surveillance plan is in place.

This study is set in the current context of AI in healthcare, therefore several limitations should be noted. First, there are relatively few published interventional trials in the field of AI for healthcare, therefore the discussion and decisions made during this study were not always supported by existing examples of completed trials. This arises from our stated aim to address the issues of poor reporting in this field as early as possible, recognising the strong drivers in the field and the specific challenges of study design and reporting for AI. As the science and study of AI evolves, we welcome collaboration with investigators to co-evolve these reporting standards to ensure their continued relevance. Second, the literature search of AI RCTs used terminology such as “artificial intelligence,” “machine learning,” and “deep learning,” but not terms such as “clinical decision support systems” and “expert systems,” which were more commonly used in the 90s for technologies underpinned by AI systems and share similar risks with recent examples.^61^ It is likely that such systems, if published today, would be indexed under “AI” or “machine learning”; however, clinical decision support systems were not actively discussed during this consensus process. Third, the initial candidate items list was generated by a relatively small group of experts consisting of steering group members and additional international experts; however, additional items from the wider Delphi group were taken forward for consideration by the consensus group, and no new items were suggested during the consensus meeting or post-meeting evaluation.

As with the CONSORT statement, the CONSORT-AI extension is intended as a minimum reporting guidance, and there are additional AI-specific considerations for trial reports which may warrant consideration (see appendix, page 2: supplementary table 2). This extension is particularly aimed at investigators and readers reporting or appraising clinical trials; however, it may also serve as useful guidance for developers of AI interventions in earlier validation stages of an AI system. Investigators seeking to report studies developing and validating the diagnostic and predictive properties of AI models should refer to TRIPOD-ML (Transparent Reporting of a Multivariable Prediction Model for Individual Prognosis or Diagnosis - Machine Learning) and STARD-AI (Standards for Reporting Diagnostic accuracy studies - Artificial Intelligence), both of which are currently under development.^32^ ^62^ Other potentially relevant guidelines are registered with the EQUATOR network, which are agnostic to study design.^63^ The CONSORT-AI extension is expected to encourage careful early planning of AI interventions for clinical trials and this, in conjunction with SPIRIT-AI, should help to improve the quality of trials for AI interventions. The development of the CONSORT-AI guidance does not include additional items within the discussion section of trial reports. The guidance provided by CONSORT 2010 on trial limitations, generalisability and interpretation were deemed to be translatable to trials for AI interventions.

There is also recognition that AI is a rapidly evolving field and there will be the need to update CONSORT-AI as the technology and newer applications for it develop. Currently most applications of AI involve disease detection, diagnosis, and triage, and this is likely to have influenced the nature and prioritisation of items within CONSORT-AI. As wider applications that use “AI as therapy” emerge, it will be important to continue to evaluate CONSORT-AI in the light of such studies. Additionally, advances in computational techniques and the ability to integrate them into clinical workflows will bring new opportunities for innovation that benefits patients. However, they may be accompanied by new challenges around study design and reporting. In order to ensure transparency, minimise potential biases, and promote the trustworthiness of the results and the extent to which they may be generalisable, the SPIRIT-AI and CONSORT-AI Steering Group will continue to monitor the need for updates.

## Web Extra

Extra material supplied by the author

Appendix: Supplementary table 1 (details of Delphi survey and consensus meeting participants) and table 2 (details of Delphi survey and consensus meeting decisions)

Supplementary fig 1: Decision tree for inclusion/exclusion and extension/elaboration

Supplementary fig 2: Checklist development process

## Notes

**The SPIRIT-AI and CONSORT-AI Working Group: **Xiaoxuan Liu,^1,2,3,4,5^ Samantha Cruz Rivera,^5,6^ David Moher,^7,8^ Melanie J Calvert,^4,5,6,9,10,11^ Alastair K Denniston,^1,2,4,5,6,12^ Hutan Ashrafian,^13,14^ Andrew L Beam,^15^ An-Wen Chan,^16^ Gary S Collins,^17^ Ara Darzi,^13,14^ Jonathan J Deeks,^10,18^ M Khair ElZarrad,^19^ Cyrus Espinoza,^20^ Andre Esteva,^21^ Livia Faes,^3,22^ Lavinia Ferrante di Ruffano,^18^ John Fletcher,^23^ Robert Golub,^24^ Hugh Harvey,^25^ Charlotte Haug,^26^ Christopher Holmes,^27,28^ Adrian Jonas,^29^ Pearse A Keane,^12^ Christopher J Kelly,^30^ Aaron Y Lee,^31^ Cecilia S Lee,^31^ Elaine Manna,^20^ James Matcham,^32^ Melissa McCradden,^33^ Joao Monteiro,^34^ Cynthia Mulrow,^35^ Luke Oakden-Rayner,^36^ Dina Paltoo,^37^ Maria Beatrice Panico,^38^ Gary Price,^20^ Samuel Rowley,^39^ Richard Savage,^40^ Rupa Sarkar,^41^ Sebastian J Vollmer,^28,42^ Christopher Yau,^28,43^

1. Academic Unit of Ophthalmology, Institute of Inflammation and Ageing, College of Medical and Dental Sciences, University of Birmingham, UK

2. Department of Ophthalmology, University Hospitals Birmingham NHS Foundation Trust, Birmingham, UK

3. Moorfields Eye Hospital NHS Foundation Trust, London, UK

4. Health Data Research UK, London, UK

5. Birmingham Health Partners Centre for Regulatory Science and Innovation, University of Birmingham, Birmingham, UK

6. Centre for Patient Reported Outcome Research, Institute of Applied Health Research, University of Birmingham, Birmingham, UK

7. Centre for Journalology, Clinical Epidemiology Program, Ottawa Hospital Research Institute, Ottawa, Canada

8. School of Epidemiology and Public Health, University of Ottawa, Ottawa, Canada

9. National Institute of Health Research Surgical Reconstruction and Microbiology Centre, University of Birmingham and University Hospitals Birmingham NHS Foundation Trust, Birmingham, UK

10. National Institute of Health Research Birmingham Biomedical Research Centre, University of Birmingham and University Hospitals Birmingham NHS Foundation Trust, Birmingham, UK

11. National Institute of Health Research Applied Research Collaborative West Midlands, Birmingham, UK

12. National Institute of Health Research Biomedical Research Centre for Ophthalmology, Moorfields Hospital London NHS Foundation Trust and University College London, Institute of Ophthalmology, London, UK

13. Institute of Global Health Innovation, Imperial College London, London, UK

14. Patient Safety Translational Research Centre, Imperial College London, London, UK

15. Harvard T.H. Chan School of Public Health, Boston, MA, USA

16. Department of Medicine, Women’s College Research Institute, Women’s College Hospital, University of Toronto, Ontario, Canada

17. Centre for Statistics in Medicine, University of Oxford, Oxford, UK

18. Institute of Applied Health Research, University of Birmingham, Birmingham, UK

19. Food and Drug Administration, Maryland, USA

20. Patient Representative

21. Salesforce Research, San Francisco, CA, USA

22. Department of Ophthalmology, Cantonal Hospital Lucerne, Lucerne, Switzerland

23. *The BMJ*, London, UK

24. *JAMA (Journal of the American Medical Association)*, Chicago, IL, USA

25. Hardian Health, London, UK

26. *New England Journal of Medicine*, Massachusetts, USA

27. Department of Statistics and Nuffield Department of Medicine, University of Oxford, Oxford, UK

28. Alan Turing Institute, London, UK

29. The National Institute for Health and Care Excellence (NICE), London, UK

30. Google Health, London, UK

31. Department of Ophthalmology, University of Washington, Seattle, Washington, USA

32. AstraZeneca, Cambridge, UK

33. The Hospital for Sick Children, Toronto, Canada

34. *Nature Research*, New York, NY, USA

35. *Annals of Internal Medicine*, Philadelphia, PA, USA

36. Australian Institute for Machine Learning, North Terrace, Adelaide, Australia

37. National Institutes of Health, Maryland, USA

38. Medicines and Healthcare products Regulatory Agency, London, UK

39. Medical Research Council, London, UK

40. PinPoint Data Science, Leeds, UK

41. The Lancet Group, London, UK

42. University of Warwick, Coventry, UK

43. University of Manchester, Manchester, UK

**Data availability:** Data requests should be made to the corresponding author and release will be subject to consideration by the SPIRIT-AI and CONSORT-AI Steering Group.
