# Supplementary material for: Reporting guidelines for clinical trial reports for interventions involving artificial intelligence: the CONSORT-AI Extension
Source: BMJ. 2020 Sep 9;370:m3164. doi: 10.1136/bmj.m3164 (PMC7490784; doi:10.1136/bmj.m3164)
Supplement: Supplementary file 2 — Supplementary fig 1: Decision tree for inclusion/exclusion and extension/elaboration [file liux059983.wf1.pdf]

Supplementary Figure 1 (CONSORT-AI): Decision tree for inclusion/exclusion and extension/elaboration.

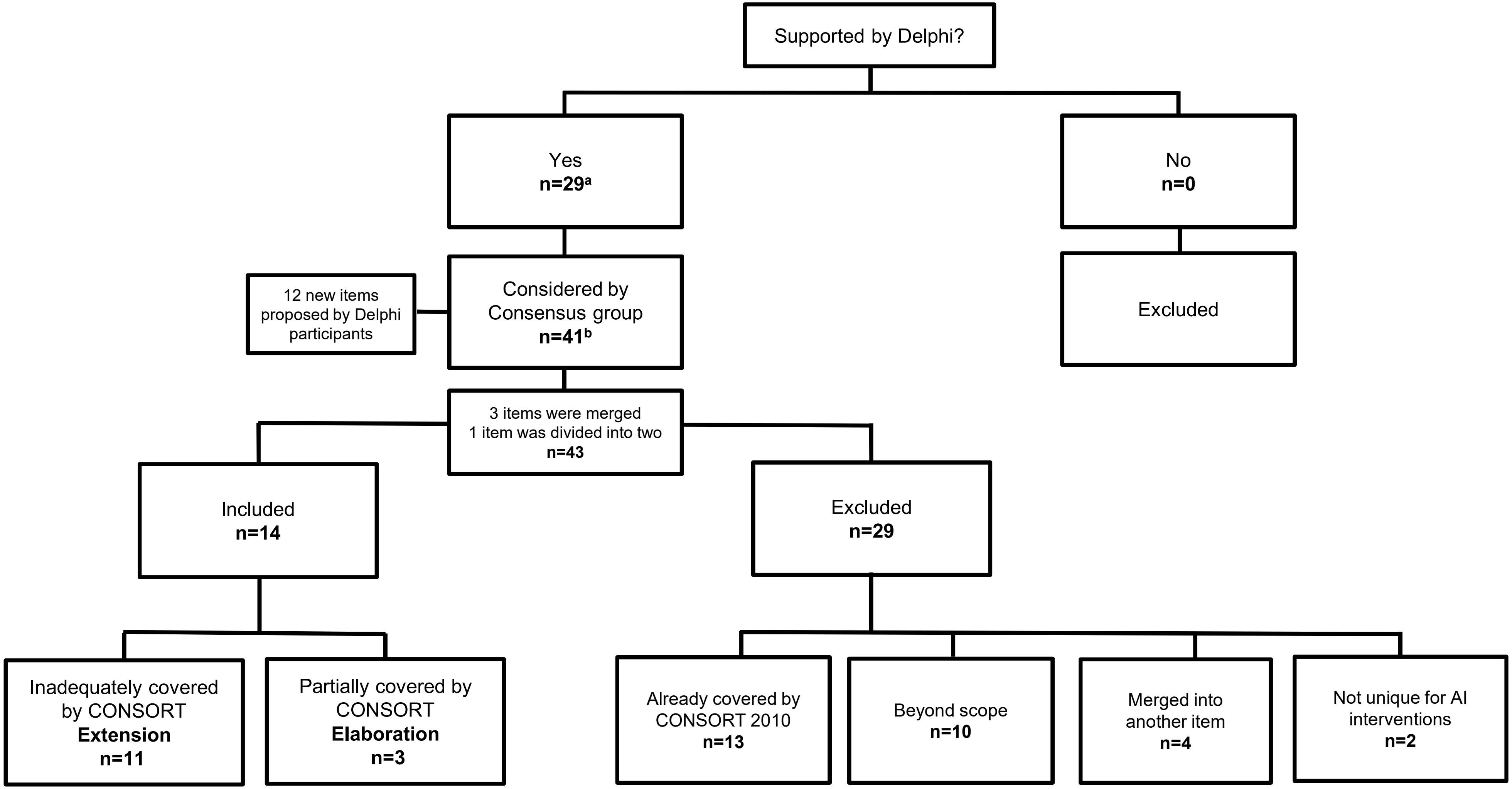

[a] Delphi exercise: inclusion criteria threshold, median score (IQR) ≥4 for (1-3) not important, (4-6) important but not critical and (7-9) important and critical items.
[b] Consensus meeting: inclusion criteria threshold, ≥80% voted included.
