# Supplementary figures and images for: Reporting guidelines for clinical trial reports for interventions involving artificial intelligence: the CONSORT-AI Extension
Source: BMJ. 2020 Sep 9;370:m3164. doi: 10.1136/bmj.m3164 (PMC7490784; doi:10.1136/bmj.m3164)

**Supplementary Figure 2:** Checklist Development Process

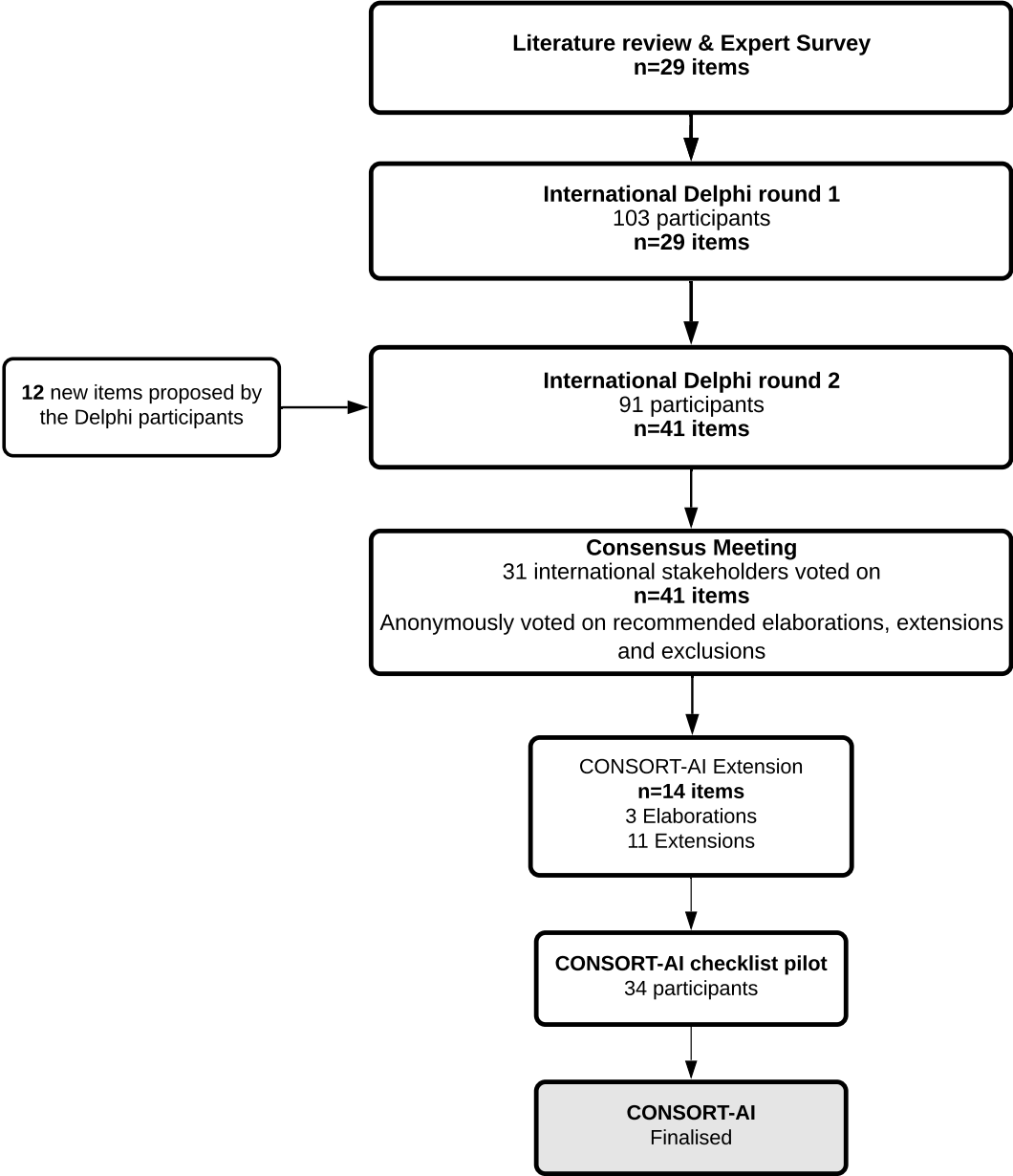

Supplement: Supplementary file 3 — Supplementary fig 2: Checklist development process [file liux059983.wf2.pdf]
